# *Lithobius* (*Ezembius*) *varioporus*, a new species from eastern China (Lithobiomorpha, Lithobiidae)

**DOI:** 10.3897/zookeys.931.47305

**Published:** 2020-04-30

**Authors:** Sujian Pei, Huiqin Ma, Haipeng Liu, Yanmin Lu, Xiaojie Hou

**Affiliations:** 1 Institute of Myriapodology, School of Life Sciences, Hengshui University, Hengshui, Hebei 053000, China Hengshui University Hengshui China; 2 Hebei Key Laboratory of Wetland Ecology and Conservation, Hengshui, Hebei 053000, China Hengshui University Hengshui China

**Keywords:** Centipede, Chilopoda, Hebei Province, myriapods

## Abstract

Lithobius (Ezembius) varioporus**sp. nov.** (Lithobiomorpha, Lithobiidae), recently discovered from Longquanguan Town, Fuping County, Baoding City, Hebei Province, China, is described. Morphologically it resembles to Lithobius (Ezembius) laevidentata Pei, Ma, Hou, Zhu & Gai, 2015 from the Xinjiang Autonomous Region, but can be easily distinguished from the latter by the Tömösváry’s organ, slightly smaller than the adjoining ocelli, no secondary sexual modifications on male tibia 14 and 15, posterior accessory spine of legs 14 and 15 present and the number of coxal pores varying considerably from three to eight. The main morphological characters of the known Chinese species of the subgenusEzembius Chamberlin, 1919 based on adult specimens are presented.

## Introduction

*Ezembius* was originally proposed as a subgenus of *Lithobius* Leach, 1814 in the family Lithobiidae by Chamberlin (1919); it accommodates a group of approximately 60 species and subspecies mostly known from Asia, with little extension into north-western North America. Known species colonise a wide range of habitats, from the Arctic and sub-Arctic regions to tropical and sub-tropical forests, from steppe and overgrazed stony areas of central Asia to Himalayan montane forests, from the seashore up to 5500 m (Himalayas) (Zapparoli and Edgecombe 2011, Qiao et al. 2018). Although the subgenus was formally proposed as new (Chamberlin 1923), according to Jeekel (2005) its name was validated in 1919 (Chamberlin 1919). *Ezembius* is characterised by antennae with ca. 20 articles; ocelli 1+4–1+20; forcipular coxosternal teeth usually 2+2; porodonts generally setiform, sometimes stout. Tergites are generally without posterior triangular projections. Female gonopods are with uni-, bi-, or tridentate claws, and 2+2–3+3 (rarely 4+4) spurs (Zapparoli and Edgecombe 2011).

The myriapod fauna of China is still poorly known and very little attention has been paid to the study of Lithobiomorpha, with only 88 species and subspecies known from the country. Altogether, 25 species of *Ezembius* have been recorded from China, but only one of them has been reported from Hebei Province (Pei et al. 2019, Qiao et al. 2019a, b). Herein, a new species recently discovered in the Hebei Province, China, is described and illustrated. Tables of the main morphological characters of Chinese *Ezembius* species are also presented.

## Materials and methods

All specimens were hand-collected under leaf litter or stones. The material was examined with the aid of a Nikon SMZ–1500 stereo microscope equipped with a drawing attachment. The colour description is based on specimens preserved in 75 % ethanol, and the body length is measured from the anterior margin of the cephalic plate to the posterior margin of the postpedal tergite. Type specimens are preserved in 75 % ethanol and deposited in the School of Life Sciences, Hengshui University, Hengshui, China (**HUSLS**). The terminology of the external anatomy follows Bonato et al. (2010).

The following abbreviations are used in the text and the tables:

**a**, anterior;

**C**, coxa;

**DaC** anterior dorsal spur of coxa;

**F**, femur;

**m**, median;

**p**, posterior;

**P**, prefemur;

**S, SS**, sternite, sternites;

**T, TT**, tergite, tergites;

**Ti**, tibia;

**To**, Tömösváry’s organ;

**Tr**, trochanter.

## Taxonomy

### Lithobiomorpha Pocock, 1895

**Lithobiidae Newport, 1844**

***Lithobius* Leach, 1814**

**Lithobius (Ezembius) Chamberlin, 1919**

#### Lithobius (Ezembius) varioporus

Taxon classificationAnimaliaLithobiomorphaLithobiidae

2ACA1DB2-5E08-59E5-A16F-29CAF25E5970

http://zoobank.org/DF87F26E-CDB7-44AE-AF12-654038A0CB93

**Figures 1–7.**
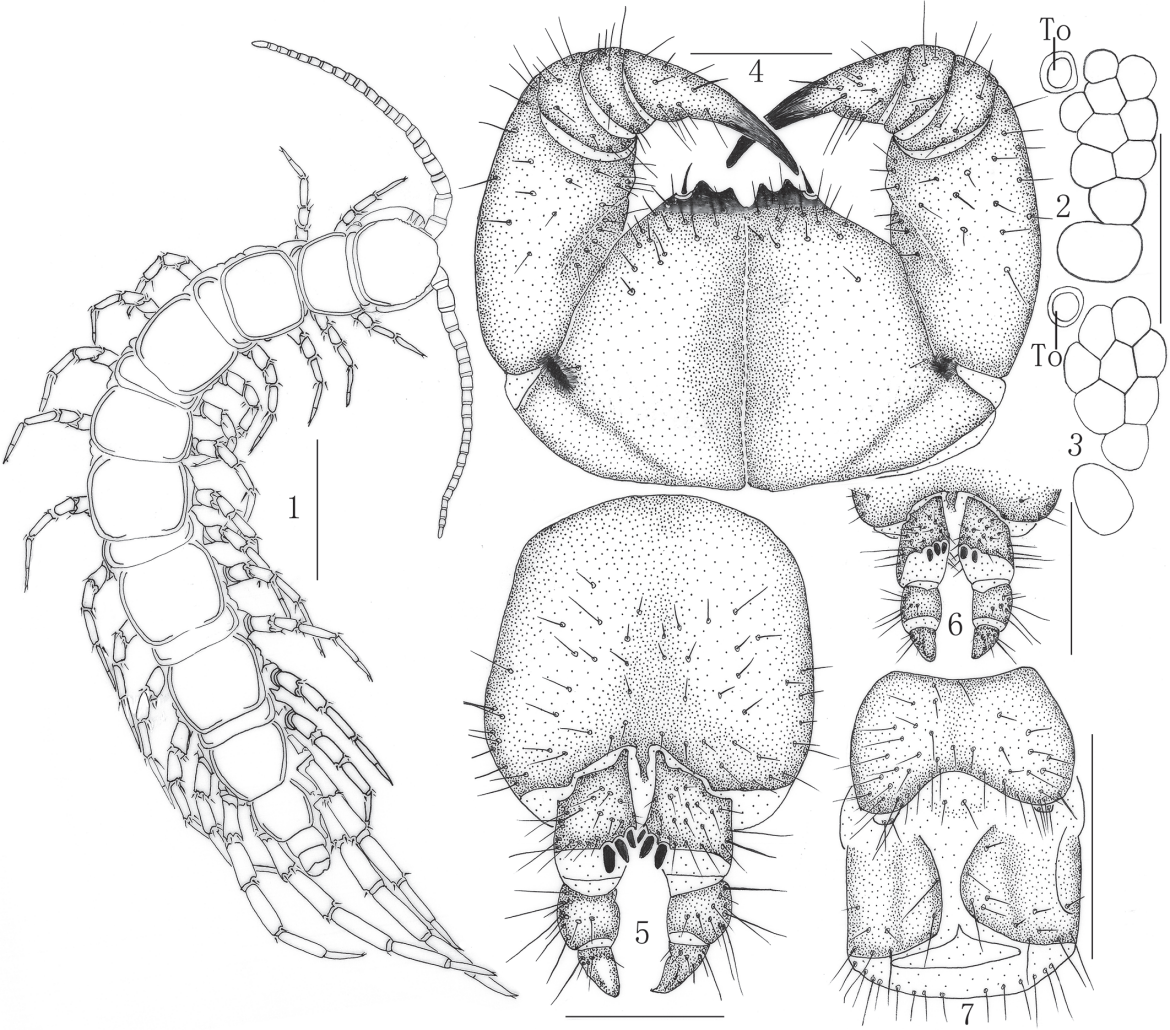
Lithobius (Ezembius) varioporus sp. nov., **1–2, 4, 7** holotype, male **1** habitus, dorsal view **2, 3** ocelli and Tömösváry’s organ **(To)**, lateral view **4** forcipulae, ventral view **5, 6** paratype, female **5** posterior segments and gonopods, ventral view **6** gonopods, ventral view **7** posterior segments and gonopods, ventral view. Scale bars: 2 mm (**1**); 250 μm (**2, 3**); 500 μm (**4–7**).

Figures 1–7 , Tables 1 , 2

##### Diagnosis.

Body length 12.4–19.1 mm, antennae composed of 20–22 articles, commonly 20 articles, 9–10 ocelli on each side of the head, arranged in three irregular rows, posterior two comparatively large ocelli; Tömösváry’s organ larger than the adjacent ocelli; commonly 2+2 forcipular coxosternal teeth, porodonts moderately slender, posterolateral to the lateral-most tooth, posterior angles of all tergites without triangular projections; 3–8 coxal pores, arranged in one row; female gonopods with 3+3 (few 3+2) moderately small coniform spurs, apical claw simple; male gonopods short and small, with three or four long setae on the terminal segment.

##### Material examined.

***Holotype***: ♂ (Fig. 1) (EV1). Body length 19.1 mm, cephalic plate 1.8 mm long, 1.7 mm wide, Heiyagou Village, Longquanguan Town, Fuping County, Baoding City, Hebei Province, China, 38°57'03.77"N, 113°48'40.70"E, 1100 m, under litter of the forest floor in a mixed coniferous broad-leaved forest, 5 August 2014, leg. S. Pei, H. Ma. ***Paratypes*** [13♀♀, 19 ♂♂] (EV1): same data as holotype. ***Other material***: 26 ♀♀, 32 ♂♂ (EV2) Liaodaobei, Longquanguan Town, Fuping County, Baoding City, Hebei Province, China, 38°50'50.12"N, 113°49'50.33"E, 1800 m, 5 August 2014, leg. S. Pei, H. Ma. 20♀♀, 21♂♂ (EV3) Wuyuezhai Mountain, Lingshou County, Shijiazhuang City, Hebei Province, China, 38°43'15.02"N, 114°08'32.62"E, 500 m, under litter of the forest floor in a mixed coniferous broad-leaved forest, 28 Sept 2014, leg. S. Pei, H. Ma.

##### Description.

Body length: 12.4–19.1 mm, cephalic plate 1.4–2.0 mm long, 1.4–1.8 mm wide.

***Colour***: antennal articles yellow-brown with blackish hue, the black gradually becomes lighter at the end of articles 6 and 7, distal-most article yellow-brown; and all tergites yellow-brown, TT 1, 3, 14, and 15 darker, pleural region pale grey with lavender hue, and sternites pale yellow-brown; basal and proximal parts of forcipules, forcipular coxosternite, SS 14 and 15 darker yellow-brown; all legs yellow-brown, distal tarsi darker.

***Antennae***: 20–22 articles, commonly 20+20 articles (Fig. 1), few specimens 20+21 or 20+22 articles; antennae article lengths are longer than wide except basal articles, which are equal to widths, distal-most article 2.9–3.2 times as long as wide; abundant setae on the antennal surface, less so on the basal articles, gradual increase in density of setae to ca. the fifth article, then more or less constant.

***Cephalic plate*** smooth, convex, slightly longer than wide; tiny setae emerging from setal sockets scattered very sparsely over the whole surface; frontal marginal ridge with shallow anterior median furrow; short to long setae scattered along the marginal ridge of the cephalic plate; lateral marginal ridge discontinuous, posterior margin continuous, straight, wider than lateral marginal ridge, the middle of the posterior edge is very slightly concaved forward (Fig. 1). Nine to ten approximate oval ocelli on each side (Figs 2, 3), domed, translucent, usually darkly pigmented, situated in three irregular rows; the posterior two ocelli comparatively large; others subequal in size. Tömösváry’s organ situated at anterolateral margin of the cephalic plate, slightly smaller than the adjoining ocelli and lying well apart from them (Figs 2, 3).

***Coxosternite*** subtrapezoidal (Fig. 1), anterior margin narrow, lateral margins slightly longer than medial margins; median diastema moderately deep, narrow U-shaped; anterior margin with 2+2 blunt triangular teeth; porodonts slender, lying posterolateral to and separated from the lateral-most tooth (Fig. 4); scattered long setae on the ventral side of coxosternite, longer setae near the dental margin.

All ***tergites*** smooth, without wrinkles, dorsum slightly convex; tiny setae emerging from setal sockets scattered sparsely over the entire surface, few long setae near the margin. Lateral marginal ridges of all tergites continuous. Posterior margin of TT 1, 3, and 5 feebly concave, posterior marginal ridges continuous, posterior margins of TT 7, 8, 10, 12, and 14 feebly concave, posterior marginal ridges discontinuous. Posterior angles of tergites generally rounded, without triangular projections. Miniscule setae scattered sparsely over the surface, two or three slightly thick and long setae on anterior and posterior angles of each tergite.

***Sternites*.** Posterior side narrower than anterior side, generally inverted trapezoidal, smooth; setae emerging from setal sockets sparsely scattered on the surface and lateral margin, 3–5 long setae on the surface of the anterior part of each sternite, two or three comparatively long setae scattered sparsely on the surface of the posterior part of each sternite.

***Legs*** robust, tarsal articulation ill-defined on legs 1–13, faint trace on ventral side, well-defined on legs 14 and 15; short to long setae sparsely scattered over the surface of prefemur, femur, tibia, and tarsus of all legs, with more setae on the tarsal surface; setae on dorsal and ventral surface of tarsus slightly longer than the anterior and posterior, one row of thicker setae regularly arranged on the medial ventral side of tibia of legs 1–13, with setae significantly reduced in legs 14 and 15, no thicker setae regularly arranged in one row on the medial ventral side of tibia. All legs with fairly long curved claws; legs 1–13 with anterior and posterior accessory spines; anterior accessory spines moderately long and slender, forming a moderately small angle with the claw, posterior accessory spines slightly more robust, forming a comparatively large angle with the claw, legs 14 and 15 only with small posterior accessory spines; legs 14 and 15 moderately thicker and longer than the anterior pairs in the female; In the female, tarsus 1 4.0–6.0 times as long as wide in legs 15, tarsus 2 ca. 73.1 %–77.3 % length of tarsus on legs 15; In the male, tarsus 1 3.3–7.3 times as long as wide in legs 15, tarsus 2 ca. 51.1 %–77.6 % length of tarsus on legs 15. Leg plectrotaxy provided in Table 1.

**Table 1.** Leg plectrotaxy of Lithobius (Ezembius) varioporus sp. nov. (based on 59 females).

Legs	Ventral					Dorsal				
	C	Tr	P	F	Ti	C	Tr	P	F	Ti
1			mp	amp	am			mp	ap	ap
2			mp	am	am			amp	ap	ap
3–10			mp	amp	am			amp	ap	ap
11			amp	amp	am			amp	ap	ap
12		m	amp	amp	am			amp	ap	ap
13		m	amp	amp	am		a	amp	p	ap
14		m	amp	amp	am		a	amp	p	p
15		m	amp	amp	a		a	amp	p	

Coxal pores 3–8, round to slightly oval, in a row; in the female, 4554, 67(8)7(8)6, 5(6)765, 6(7)776, 66(7)65, in the male, 66(7)7(6)5(4), 565(6)3, coxal pore field set in a relatively shallow groove, the coxal pore-field fringe with a prominence; prominence with 10–15 short to moderately long setae sparsely scattered over the surface.

***Female*** S 15 anterior margin broader than posterior, generally inverted trapezoidal, posteriomedially straight, colour usually yellow-brown; short to long sparse setae evenly scattered on surface; surface of the lateral sternal margin of genital segment well chitinised, posterior margin of genital sternite deeply concave between condyles of gonopods, except for a small, median approximately rhombic-shaped bulge; relatively long setae sparsely scattered over ventral surface of the genital segment. Gonopods (Figs 5, 6): first article fairly broad, bearing 22–26 moderately long setae, arranged in five irregular rows; generally with 3+3 (3+2 in only two specimens) moderately long and slender, coniform spurs, inner spur slightly smaller than the outer, dorsolateral setae absent; second article with 12–16 long setae, arranged in three irregular rows, 9–12 stout setae on the dorsal side; third article with five or six comparatively long setae, arranged in two irregular rows, four or five stout setae on the dorsal side; third article with a simple broad apical claw (Figs 5, 6).

***Male*** S 15 posterior margin narrower than anterior, posteriomedially straight, sparsely covered with long setae on the surface; sternite of genital segment smaller than in female, usually well sclerotised, posterior margin deeply concave between the gonopods, without median bulge; long setae sparsely scattered on the ventral surface of the genital segment, fringed with longer setae along the posterior margin; gonopods short, appearing as a small hemispherical bulge, with three or four long setae, apically slightly sclerotised (Fig. 7).

##### Habitat.

The specimens studied here were collected from a mixed coniferous broad-leaved forest at ca. 500–1800 m above sea level, in moderately moist habitats under roadside stones and litter of the forest floor.

##### Etymology.

The specific epithet *varioporus* refers to the coxal pore numbers varying considerably from three to eight.

##### Discussion.

The new species is morphologically close to L. (E.) laevidentata Pei, Ma, Hou, Zhu & Gai, 2015 and to L. (E.) tetraspinus Pei, Lu, Liu, Hou & Ma, 2018, both from the Xinjiang Uygur Autonomous Region, China, with which it shares 20–22 antennal articles, 9–10 ocelli arranged in three irregular rows, the posterior two ocelli comparatively large, 3+3 spurs on female gonopods, no anterior accessory spines on legs 15.

However, the new species can be easily distinguished from L. (E.) laevidentata by the size of the Tömösváry’s organ, slightly smaller than the adjoining ocelli, rather than subequal to the largest ocellus as in L. (E.) laevidentata. The new species has no secondary sexual modifications on the male 15 tibia compared to L. (E.) laevidentata, in which a distinct and shallow dorsal furrow is present on the same leg; moreover, in the new species legs 14 and 15 bears a small accessory spines only on the posterior side vs. both anterior and posterior accessory spines are present on legs 14, and only with posterior accessory spines present on legs 15 in L. (E.) laevidentata.

The new species can be easily distinguished from L. (E.) tetraspinus by the Tömösváry’s organ, slightly smaller than the adjoining ocelli in contrast to subequal in size to adjoining ocelli in L. (E.) tetraspinus. Moreover, the new species has no secondary sexual modifications on the leg 15 male tibia vs. the dorsal sulci on the femur in L. (E.) tetraspinus. In the new species, legs 14 and 15 bear small accessory spines only in the posterior side instead of both anterior and posterior accessory spines present on legs 14, and lacking accessory spines on legs 15 in L. (E.) tetraspinus. The new species must be also easily distinguished from the other Lithobius (Ezembius) species to date known from China by the coxal pore number, varying considerably from three to eight, not only among specimens, but also in the same individual.

To assist in the identification of the Lithobius species of the subgenus Ezembius from China, the main morphological characters based on adult specimens are presented in Table 3.

**Table 2.** Leg plectrotaxy of Lithobius (Ezembius) varioporus sp. nov. (based on 73 males).

Legs	Ventral					Dorsal				
	C	Tr	P	F	Ti	C	Tr	P	F	Ti
1			mp	amp	am			mp	ap	a
2–11			mp	amp	am			amp	ap	ap
12			amp	amp	am		a	amp	ap	ap
13		m	amp	amp	am		a	amp	p	ap
14		m	amp	amp	am		a	amp	p	p
15		m	amp	am(p)	a		a	amp	p	

Letters in brackets indicate variable spines (absent in 5 specimens).

**Table 3.** Range and main morphological characters of the known Chinese species of subgenus Lithobius (Ezembius) Chamberlin, 1919.

**Characters**	***L. anabilineatus***	***L. anasulcifemoralis***	***L. bidens***	***L. bilineatus***	***L. chekianus***	***L. datongensis***	
Authorities	Ma et al. 2015	Ma et al. 2013	Takakuwa 1939	Pei et al. 2014	Chamberlin and Wang 1952	Qiao et al. 2018	
Distribution	China S (Guangxi)	China S (Guangxi)	China S (Taiwan)	China S (Guangxi)	China S (Zhengjiang and Taiwan)	China NW (Qinghai Province)	
Body length (mm)	11.9–12.1	10.1–12.3	15.0	9.0–9.1	16.0	12.3–14.2	
Number of antennal articles	23+23 articles in female, unkown in male	19+19–24+24, commonly 20+20	20–21	two specimens with 20+21, one specimen with 20+23	20+20	20+20	
Number, arrangement and shape of the ocelli	5 – 6, in 2 rows	6, in 3 rows	7	5–6, in 2 rows	5, in 3 rows	10, in 3 rows	
Posterior ocellus	round, large	oval to round, large	comparatively large	oval to rounded	oval to round, comparatively large	comparatively large	
Seriate ocelli	subequal, all ocelli domed, translucent, usually darkly pigmented	one near ventral margin moderately small, others almost equal	not reported	subequal, all ocelli domed, translucent, usually darkly pigmented	not reported	not reported	
Tömösváry’s organ	round, smaller than adjoining ocelli	moderately large, rounded, slightly larger than adjoining ocelli	at most same size as one ocellus	slightly larger than adjoining ocelli	not reported	slightly larger than nearest ocellus	
Number and arrangement of coxosternal teeth	2+2, subtriangular	2+2, moderately blunt	2+2	2+2, slightly triangular	2+2	2+2 slightly acute	
Porodont	long, lying posterolateral to lateral-most teeth	slender, lying posterolateral to lateral-most tooth, their base moderately bulged	moderately long	thick and long, lying posterolateral to lateral-most tooth	not reported	setiform porodonts separated from lateral tooth laterally	
**Characters**	***L. anabilineatus***	***L. anasulcifemoralis***	***L. bidens***	***L. bilineatus***	***L. chekianus***	***L. datongensis***	
Tergites	smooth, backside slightly hunched	smooth	not reported	smooth, slightly hunched behind	not reported	almost smooth	
Number of coxal pores	3–5, female 4454, 3554; male 4443, 4453	3–6, usually 4663, 5654, 5553, 5563 and 5565	5 (6) 555	usually females 4554, 5565; males 4553, 4454	6655 or 7665	4655 and 5575. Coxal pores 4654 and 4554 in male	
Shape of coxal pores	round or slightly ovate	round or slightly ovate	round	ovate	not reported	rounded	
Tarsus 1–tarsus 2 articulation on legs 1–13	not well-defined	not well-defined	Well-defined	not well-defined	not reported	distinct	
Male 14^th^ legs	Obvious, thicker and stronger than other legs	markedly thicker and stronger than 1–13 legs, thicker and stronger than female	not reported	distinctly thick and strong	not reported	not reported	
Male 15^th^ legs	obvious thicker and stronger than other legs	markedly thicker and stronger than 1–13 legs, thicker and stronger than female	not reported	distinctly thick and strong	not reported	not reported	
Dorsal sulci on male 14^th^ legs	absent	absent	not reported	with two, shallow longitudinal sulci	not reported	not reported	
Dorsal sulci on male 15^th^ legs	two distinct, shallow, dorsal sulci on femur and tibia	with a distinct, shallow, dorsal sulci on tibia	not reported	with two, shallow longitudinal sulci	not reported	not reported	
DaC spure	on 14^th^–15^th^ legs	on 14^th^–15^th^ legs	absent	on 4^th^–15^th^ legs	on 14^th^–15^th^ legs	on 12^th^–15^th^	
14^th^ accessory spine	anterior accessory spine reduced in size, only half length of posterior accessory spine	absent	not reported	anterior accessory spine absent	present	present	
15^th^ accessory spine	absent	absent	not reported	anterior accessory spine absent	present	anterior accessory spine absent	
Number and shape of spurs on female gonopods	2+2 moderately small, blunt, coniform spurs, inner spur slightly smaller than the outer	2+2 moderately blunt, with conical spurs, inner spur slightly smaller	3+3 or 4+4, sharp	2+2 moderately small, blunt, coniform spurs, inner spur slightly smaller than outer one	not reported	2+2 moderately large, coniform spurs	
Dorsal side of second article of female gonopods	with one spine lying dorsally on its external margin	no striking features	not reported	with three short, robust setae lying dorsally on its external margin	not reported	5-6 setae and five long curved spines	
Apical claw of female gonopods (and lateral denticles)	simple, small subtriangular teeth in the inner	apical claw dimidiate	simple, small sharply teeth in the inner	apical claw bipartite, and its inner aspect broader	not reported	undivided, bearing a small triangular protuberance on ventral side	
Male gonopods	short and small bulge, with one to two long setae, apically slightly sclerotised	with a small bulge, without setae and apically less sclerotised	hemispherical, with two long setae	short and small bulge, having a long seta, apically slightly sclerotised	not reported	a hemispherical bulge, with three setae	
**Characters**	***L. dulanensis***	***L. gantoensis***	***L. giganteus***	***L. insolitus***	***L. irregularis***	***L. laevidentata***	
Authorities	Qiao et al. 2019	Takakuwa and Takashima 1949	Eason 1986	Eason 1993	Takakuwa and Takashima 1949	Pei et al. 2015	
Distribution	China NW (Qinghai Province)	China NW (Shanxi)	China N (Inner Mongolia Autonomous region)	China S (Hong Kong)	China W (Shanxi)	China NW (Xinjiang Uygur)	
Body length (mm)	20.5	9.0	15.0–50.0	10.0–11.5	12.0	9.6–13.3	
Number of antennal articles	20–21	20–23	20+20	18+18–19+19	20+20	19+19–21+21 commonly 20+20	
Number, arrangement and shape of the ocelli	11–12, in 3 rows	6, in 2 rows	6–10, in 2–3 rows	6–8, in 2 rows	7, in 2 rows	8–10, in 3 rows	
**Characters**	***L. dulanensis***	***L. gantoensis***	***L. giganteus***	***L. insolitus***	***L. irregularis***	***L. laevidentata***	
Posterior ocellus	oval to rounded, comparatively large	oval to round, comparatively large	oval to round, comparatively large	oval to round, comparatively large	round, comparatively large	posterior two ocelli bigger than seriate ocelli	
Seriate ocelli	the second row smaller than the first, the third smallest	comparatively large	not reported	not reported	subequal	other seriate ocelli slightly larger than ocelli adjoining ventrally	
Tömösváry’s organ	Slightly smaller than the adjoining ocelli	subequal in size to adjoining medium large ocelli	slightly smaller than adjoining ocelli	slightly smaller than adjoining ocelli	same size as largest ocellus	subequal in size to adjoining ocelli	
Number and arrangement of coxosternal teeth	2+2 moderately robustteeth	2+2, approximately sharp, small	2+2	2+2, approximately sharp, small	2+2, small	2+2, approximately blunt	
Porodont	Slender lying posterolateral to the most lateral tooth	not reported	not reported	slender, lying posterolateral to lateral tooth, their base slightly bulged	long, their base slightly bulged	thick and long, lying posterolateral to lateral-most teeth	
Tergites	Smooth and all posterior angles rounded without projections	smooth, without wrinkles	smooth, with slightly wrinkles	T1 smooth, other with wrinkles	smooth	smooth, without wrinkles, backside slightly hunched	
Number of coxal pores	5667 or 5666	3333	3333, 4554, 4555, 4565, 5565 or 5566	3–6, male 3443; female 4454, 4555, 5555, 5565	3–10, female 3–6 in 12^th^ leg, 4–6 in 13^th^ leg, 7–10 in 14^th^ and 15^th^ leg	2–5, female commonly 4555, 4554, sometime 3454, 3455, 3343. male commonly 2332, 2333, sometime 3444, 3333	
Shape of coxal pores	Circularor slightly ovate	round	round	round	round	round or slightly ovate	
Tarsus 1–tarsus 2 articulation on legs 1–13	fused	not reported	Well-defined	not defined	Well-defined	not well-defined	
Male 14^th^ legs	longer and thicker than legs 1–13	not reported	not reported	distinctly thick and strong	not reported	remarkably thicker and stronger	
Male 15^th^ legs	longer and thicker than legs 1–13	not reported	not reported	distinctly thick and strong, with dark zones on dorsal of tibia	not reported	markedly thicker and stronger	
Dorsal sulci on male 14^th^ legs	absent	not reported	not reported	absent	not reported	absent	
Dorsal sulci on male 15^th^ legs	absent	not reported	not reported	absent	not reported	with a distinct, shallow, dorsal sulci on the tibia	
DaC spure	on 11^th^–15^th^	absent	on 12^th^–15^th^ legs (on 11^th^ and 12^th^ legs sometimes present)	absent	on 13^th^–15^th^ legs	on 12^th^–15^th^ legs	
14^th^ accessory spine	anterior accessory spines absent	present	present	not reported	not reported	present	
15^th^ accessory spine	absent	present	absent	absent	not reported	anterior accessory spines absent	
Number and shape of spurs on female gonopods	2+2 moderately small coniform spurs	1+1, conical spurs	2+2	3+3, coniform spurs	2+2 or 2+3, moderately small, blunt, coniform spurs	3+4, or 4+4 small, blunt, coniform spurs, commonly with 3+3, inner spur smaller than outer one	
Dorsal side of second article of female gonopods	with six dorsolateral setae	not reported	with eight spines in two irregular rows lying dorsally on its external margin	not reported	not reported	with three long setae lying dorsally on its anterior external margin	
Apical claw of female gonopods (and lateral denticles)	unidentate, curved	simple	simple	simple	simple and broad	simple and broad	
Male gonopods	small, one-segmented, with two long setae, apically slightly chitinized, flat	not reported	not reported	not reported	not reported	small bulge, with one to two long setae apically slightly sclerotised	
**Characters**	***L. longibasitarsus***	***L. lineatus***	***L. mandschreiensis***	***L. maqinensis***	***L. multispinipes***	***L. parvicornis***	
Authorities	Qiao et al. 2018	Takakuwa 1939	Takakuwa 1940	Qiao et al. 2019b	Pei et al. 2016	Zapparoli 1991	
Distribution	China NW (Qinghai)	China S (Taiwan)	China (Taiwan, Sichuan, Jiangsu, Heilongjiang, Jilin, Liaoning)	China NW (Qinghai)	China NW (Xinjiang Uygur)	China S (Taiwan)	
**Characters**	***L. longibasitarsus***	***L. lineatus***	***L. mandschreiensis***	***L. maqinensis***	***L. multispinipes***	***L. parvicornis***	
Body length (mm)	17.0–18.0	18.0	22.0–23.0	13.10–14.60	11.6–22.6	16.0	
Number of antennal articles	20+20	19+19–21+21	20–28	20+20	commonly 20+20, (three specimens with 20+21, one specimen with 20+26 of 134 specimens)	20+20, 21+21	
Number, arrangement and shape of the ocelli	11, in 3 rows	8–11, in 3 rows	9–13, in 3 rows	9–10, in 3 rows	8, in 3 rows	3–4, in 1 or 2 rows	
Posterior ocellus	posterior ocellus largest	comparatively small	comparatively large	the most posterior ocellus largest	two ocelli large, oval to rounded	comparatively large	
Seriate ocelli	not reported	not reported	same size	the ocelli of the bottom row small	two near ventral margin moderately small, others almost equal	not reported	
Tömösváry’s organ	smaller than adjacent ocelli	same size as adjoining ocelli	larger than adjoining ocelli	almost the same size as adjacent ocelli	slightly smaller than adjoining ocelli	not reported	
Number and arrangement of coxosternal teeth	3+2 blunt nipple-like teeth	2+2, comparatively large	2+2, small and sharp	2 + 2	3+3, slightly triangular	2+2	
Porodont	thick and strong separated from lateral tooth ventrolaterally	long and strong	lying posterolateral to lateral-most tooth	setiform porodonts on small knobs	thick and long, lying posterolateral to lateral-most tooth	lying posterolateral to the lateral-most teeth	
Tergites	all smooth, without wrinkles	smooth	smooth, without wrinkles	smooth, never rugose	smooth, without wrinkles and slightly hunched behind	smooth	
Number of coxal pores	6555	6–7, usually 66(7)6	776(7)5(6)	6666	3–5, 4555, 5555, 4444, 4455 (females) and 4444, 3344 (males)	3334	
Shape of coxal pores	circular	round to ovate	round or ovate	round and uni-seriate, the most proximal pore on 15th coxae minute	round to ovate	not reported	
Tarsus 1–tarsus 2 articulation on legs 1–13	well-defined	well-defined	well-defined	unipartite tarsi	well-defined	not reported	
Male 14^th^ leg	moderately thicker and longer	not reported	not reported	longer and thicker than 1–13	thick and strong	not reported	
Male 15^th^ leg	moderately thicker and longer	not reported	not reported	longer and thicker than 1–13	thick and strong	not reported	
Dorsal sulci on male 14^th^ legs	absent	absent	not reported	not reported	absent	not reported	
Dorsal sulci on male 15^th^ legs	absent	not reported	not reported	not reported	absent	not reported	
DaC spure	on 13^th^–15^th^ legs, 12^th^ sometimes present	on 14^th^–15^th^ legs	on 12^th^–15^th^ legs	on 12^th^–15^th^ legs, 11^th^ sometimes present	on 11^th^–15^th^ legs, 9^th^–10^th^ sometimes present	not reported	
14^th^ accessory spine	present	present	not reported	posterior accessory spurs present	present	not reported	
15^th^ accessory spine	absent	present	not reported	absent	absent	not reported	
**Characters**	***L. longibasitarsus***	***L. lineatus***	***L. mandschreiensis***	***L. maqinensis***	***L. multispinipes***	***L. parvicornis***	
Number and shape of spurs on female gonopods	2+2 moderately long, bullet-shaped spurs inner spur slightly smaller and more anterior than outer one	3+3 moderately sharp, slender conical spurs	3+3, same size	2+2 moderately small, coniform spurs, inner spur smaller	2+2, blunt, coniform spurs, with inner spur smaller than outer one	2+2	
**Characters**	***L. longibasitarsus***	***L. lineatus***	***L. mandschreiensis***	***L. maqinensis***	***L. multispinipes***	***L. parvicornis***	
Dorsal side of second article of female gonopods	three long setae along dorsolateral ridge	not reported	not reported	not reported	with 3–4 long setae and 5–6 spines lying dorsally on its external margin	not reported	
Apical claw of female gonopods (and lateral denticles)	simple, having small triangular protuberance on ventral side	simple	simple	unidentate, curved with a small triangular protuberance on ventral side	simple	simple	
Male gonopods	small, semicircular article with 3-5 seta on its surface	hemispherical bulge,	without setae	small, undivided, oblique apically, with 2 setae	hemispherical bulge, having a long seta, and apically slightly sclerotised	not reported	
**Characters**	***L. polyommatus***	***L. rhysus***	***L. sulcipes***	***L. sulcifemoralis***	***L. tetraspinus***	***L. varioporus***	***L. zhui***
Authorities	Qiao et al. 2019	Attems 1934	Attems 1927	Takakuwa and Takashima 1949	Pei et al. 2018	This paper	Pei et al. 2011
Distribution	China NW (Tibet)	China S (Fujian and Taiwan)	China S (Taiwan)	China W (Shanxi)	China NW (Xinjiang Uygur)	China E (Hebei)	China NW (Xinjiang Uygur)
Body length (mm)	16.10 –18.30	15.0	Not reported	12.0	9.6–13.3	12.4–19.1	8.1–15.0
Number of antennal articles	20+20	20+20 in female, 20+21 in male	19–22	20+20	19–22, commonly 20	20–22	20–24, commonly 20
Number, arrangement and shape of the ocelli	14, in 3 rows	8, in 4 rows	7, in 2 rows	6	8–10, in 3 rows	9–10, in 3 rows	10–13, in 3–4 rows
Posterior ocellus	posterior ocellus comparatively large	comparatively large	comparatively large	all ocelli same size	two ocelli comparatively large	posterior two ocelli comparatively large	comparatively large
Seriate ocelli	almost equal	not reported	not reported	same size	the adjoining Tömösváry organ slightly small	others subequal in size	dorsal ones moderately large, those near ventral margin of ocellar field moderately small, others of moderate size
Tömösváry’s organ	moderately smaller than the adjoining ocelli	not reported	not reported	same size as ocelli	subequal in size to adjoining ocelli	slightly smaller than the adjacent ocelli	slightly larger than adjoining ocelli
Number and arrangement of coxosternal teeth	2 + 2 subtriangular slightly acute teeth	2+2	2+2	2+2, small and sharp	2+2 subtriangular slightly acute	2+2 blunt triangular teeth	2+2 moderately small and pointed
Porodont	thick and strong, just posterolateral and separated from the lateral tooth	not obvious	not reported	slender and long	thick and strong, just posterolateral and separated from lateral tooth	slender, lying posterolateral to and separated from the lateral-most tooth	moderately thick in basal, moderately pointed, just posterolateral to lateral tooth
Tergites	smooth without wrinkles	with shallow wrinkles	Smooth, posterior angles slightly triangular in T14	not reported	smooth, without wrinkles, dorsum slightly convex	smooth, without wrinkles, dorsum slightly convex	smooth, without wrinkles, backside slightly hunched
Number of coxal pores	4–7, 5676, 5666 (females) 5565, 4554 (males)	6554	4554	5555	usually 4555, 4554, rarely 3454, 3455, 3343 in females and usually 2332, 2333, rarely 3444, 3333 in males	in the female, 4554, 67(8)7(8)6, 5(6)765, 6(7)776, 66(7)65, in the male, 66(7)7(6)5(4), 565(6)3	2–4, 3444, 3344, 3443, 3333 in female, and 3443, 2343, 2433, 2333 in male.
Shape of coxal pores	round or slightly oval	round	round	round	round or slightly oval	round to slightly oval	round or slightly ovate
Tarsus 1–tarsus 2 articulation on legs 1–13	ill-defined	not reported	well-defined	well-defined	ill–defined	well-defined	well–defined
Male 14^th^ legs	slightly thicker in the female, significantly thicker and stronger in the male	not reported	not reported	thick and strong	significantly thicker and stronger	moderately thicker and longer	moderately thicker and stronger
**Characters**	***L. polyommatus***	***L. rhysus***	***L. sulcipes***	***L. sulcifemoralis***	***L. tetraspinus***	***L. varioporus***	***L. zhui***
Male 15^th^ legs	slightly thicker in the female, significantly thicker and stronger in the male	femur and tibia thicker	femur and tibia thicker	thick and strong	significantly thicker and stronger	moderately thicker and longer	thicker and stronger, with a circular protuberance on distal end of tibia
Dorsal sulci on male 14^th^ legs	with a longitudinal discontinuous shallow and narrow groove on dorsal side of tibia, and a faintly black vertical line at the bottom on dorsal side	not reported	present on femur	present on femur and tibia	absent	absent	absent
Dorsal sulci on male 15^th^ legs	with a longitudinal discontinuous shallow and narrow groove on dorsal side of tibia, and a faintly black vertical line at the bottom on dorsal side	not reported	present on femur and tibia	present on femur and tibia	present on femur	absent	absent
DaC spure	on 11^th^–15^th^ legs	on 15^th^ legs present	on 15^th^ legs present	absent	on 12^th^–15^th^ legs	on 12^th^–15^th^ legs	on 13^th^–15^th^ legs, 12^th^ sometimes present
14^th^ accessory spine	present	not reported	not reported	not reported	present	anterior accessory spine absent	present
15^th^ accessory spine	absent	absent	not reported	not reported	absent	anterior accessory spine absent	absent
Number and shape of spurs on female gonopods	2 + 2 moderately long and slender, bullet-shape spurs	2+2, slender	2+2, thick spurs	2+2, strong, long and sharp	3+3, few 3+4, only one 4+4 coniform spurs	3+3 (seldom 3+2) moderately long and slender, coniform	2+2 moderately long, coniform spurs, inner spur slightly smaller and more anterior than outer
Dorsal side of second article of female gonopods	9 long setae lying dorsally on the posterior part of the external margin	not reported	not reported	not reported	3 long setae and four short, robust spines lying dorsally on posterior part of external margin	no setae and spines	three spurs arranged in one irregular row on dorsal terminal part
Apical claw of female gonopods (and lateral denticles)	simple	simple	dimidiate	simple	simple, with a very small subtriangular blunt denticle on inner margin	simple	broad, and tridentate
Male gonopods	short, apically slightly sclerotized, appearing as a small hemispherical bulge with 2 long setae	not reported	not reported	not reported	small hemispherical bulge, with 1–2 long setae	short, small hemispherical bulge, with 1–3 long setae, apically slightly sclerotized	small bulge, with 1–2 long setae on surface, and terminal slightly sclerotised

## Supplementary Material

XML Treatment for Lithobius (Ezembius) varioporus
